# Ultrastable and High-Performance Silk Energy Harvesting Textiles

**DOI:** 10.1007/s40820-019-0348-z

**Published:** 2019-12-30

**Authors:** Chao Ye, Shaojun Dong, Jing Ren, Shengjie Ling

**Affiliations:** School of Physical Science and Technology, ShanghaiTech University, 393 Middle Huaxia Road, Shanghai, 201210 People’s Republic of China

**Keywords:** Silk, Energy harvesting textile, Co-wrapped yarn, Triboelectric nanogenerator

## Abstract

Energy harvesting textiles (EHTs) have attracted much attention in wearable electronics and the internet-of-things for real-time mechanical energy harvesting associated with human activities. However, to satisfy practical application requirements, especially the demand for long-term use, it is challenging to construct an energy harvesting textile with elegant trade-off between mechanical and triboelectric performance. In this study, an energy harvesting textile was constructed using natural silk inspired hierarchical structural designs combined with rational material screening; this design strategy provides multiscale opportunities to optimize the mechanical and triboelectric performance of the final textile system. The resulting EHTs with traditional advantages of textiles showed good mechanical properties (tensile strength of 237 ± 13 MPa and toughness of 4.5 ± 0.4 MJ m^−3^ for single yarns), high power output (3.5 mW m^−2^), and excellent structural stability (99% conductivity maintained after 2.3 million multi-type cyclic deformations without severe change in appearance), exhibiting broad application prospects in integrated intelligent clothing, energy harvesting, and human-interactive interfaces.

**Electronic supplementary material:**

The online version of this article (10.1007/s40820-019-0348-z) contains supplementary material, which is available to authorized users.

## Article Highlights

- An energy harvesting textile with an elegant trade-off of mechanical and triboelectric performance was constructed by hierarchical structural design.
- Modified rotor co-spinning technique was developed to produce triboelectric yarns in large scale.
- Mass-producible energy harvesting textiles show ultrahigh stability withstands millions of multi-type cyclic deformations and various applications.

**Electronic supplementary material:**

The online version of this article (10.1007/s40820-019-0348-z) contains supplementary material, which is available to authorized users.

## Graphical abstract

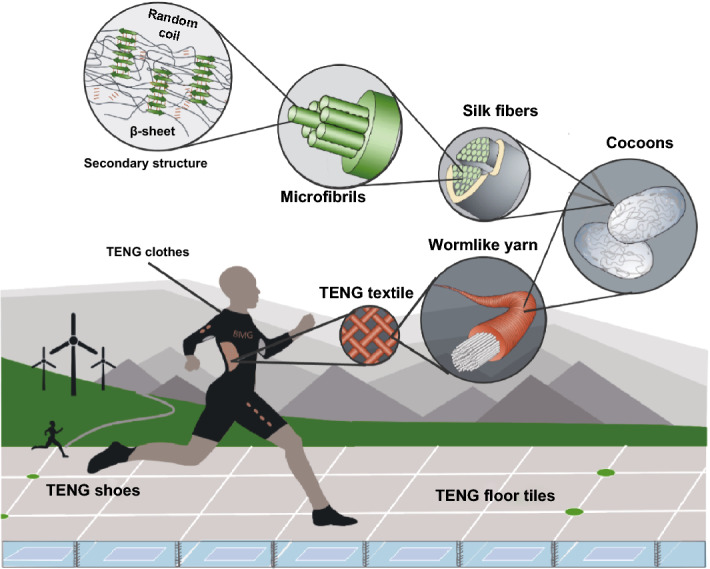

## Introduction

Integrated intelligent clothing has received much attention because of the urgent need of constantly monitoring human body conditions to prevent disease, providing extra protection, and other promising applications such as human enhancement and customized high fashion [1, 2]. With the ultra-rapid development of various wearable devices, power supply for intelligent clothing remains a challenge. Therefore, triboelectric nanogenerators (TENGs) have been developed for real-time mechanical energy harvesting associated with human activities [3–7]. Compared with conventional power supply systems such as electromagnetic generators, thermoelectric generators [8], and solar cells [9], TENGs have the advantages of lightweight materials, mass production, wide choice of materials, and efficient low-frequency energy harvesting [10–15]. Besides, TENGs show wide applications, such as sustainable micropower sources; active sensors for medical, infrastructure, environmental monitoring, and human–machine interfacing; low-frequency water energy harvesting; and power sources for high-voltage instruments [7].

Integration of polymer fibers/fabrics with conductive nanomaterials such as carbon nanomaterials and metal nanomaterials [16–20] is one of the most widely used strategies for fabricating TENG-based energy harvesting textiles (EHTs) and has improved the energy harvesting efficiency. In most of these EHTs, the combination of polymers and inorganic nanomaterials is often achieved through solution coating or atomic deposition [21–24]. The mechanical robustness and structural stability of these EHTs are still unable to satisfy the requirements of practical applications, especially demands for long-term use. For example, EHTs produced by coating and atomic deposition generally cannot tolerate daily washing [17]. In practice, other wearable devices such as fiber-shaped energy storage devices and flexible sensors also suffer from incompatibility of mechanical robustness, structural stability, and high functional performance. For example, most functional fibers (e.g., fiber-shaped electrodes [25–27]), wearable devices (e.g., strain/press sensors [28, 29]), and smart textiles (e.g., triboelectric textiles [30]) can only tolerate tens of thousands of single-form deformations. Such a performance is far below the required clothing performance in practical applications. In general, a practical electronic garment should be able to withstand millions of times of friction and deformation, even if it is worn for 1 year only. In this regard, natural materials such as silk and dactyl club provide inspiration because most of them are required to be constitutionally stable, mechanically strong, and tough to satisfy the need of versatile functional long-term uses [31–34]. The nature uses hierarchical design to construct materials. This strategy offers multiscale opportunities to govern the “internal” structure–property relationship of materials, thereby achieving “external” functions or requirements.

**Fig. 1:**
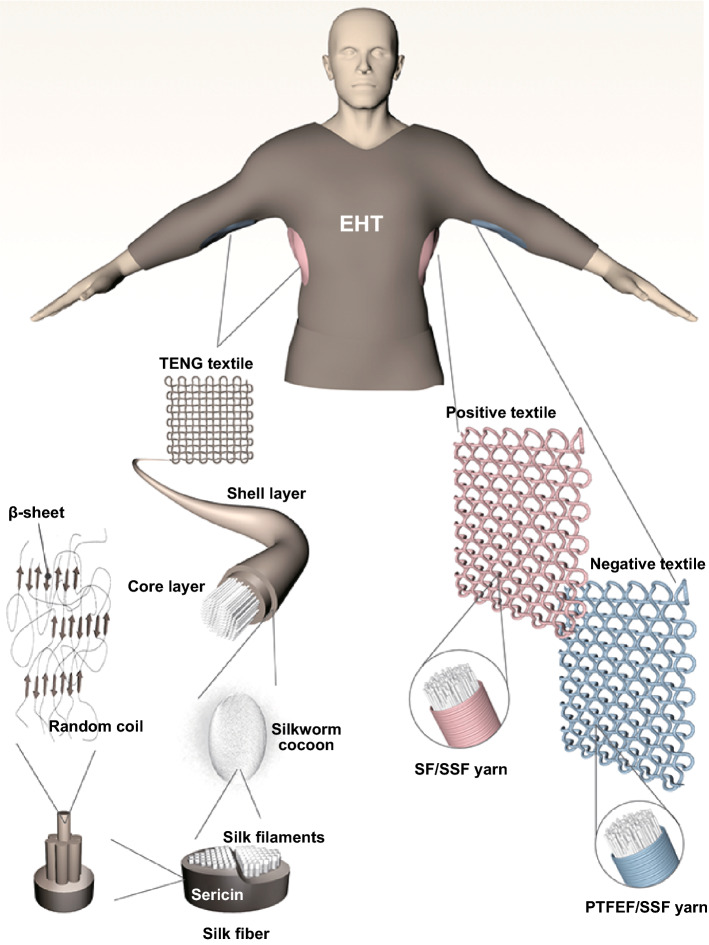
Schematic illustration of the SF/PTFEF EHT

In this study, TENG-based EHTs were constructed using hierarchical designs to improve their overall performance (Fig. 1) including high mechanical strength and flexibility, long-term stability, outstanding processability, wearability, as well as useful triboelectric performance. Among them, mechanical strength and ductility are crucial for long-term stability, processability, and wearability. Accordingly, the trade-off between structure, mechanics, and triboelectric properties should be considered. A de novo and mass-producible textile technology was developed to fabricate these predesigned EHTs using silk fiber (SF), polytetrafluoroethylene fiber (PTFEF), and stainless steel fiber (SSF) as the starting materials. The resulting SF/PTFEF EHT features unique hierarchical structures and thus provide multiscale opportunities to optimize the mechanical and triboelectric performance of the final system, including a high tensile strength of 237 ± 13 MPa and toughness of 4.5 ± 0.4 MJ m^−3^ for single yarns, high power output of 3.5 mW m^−2^, and ultrahigh stability with a performance of almost no difference after 2.3 millions of multi-type cyclic deformations. The designed EHTs show broad application prospects in wearable electronics, motion tracking, artificial intelligence, and human-interactive interfaces.

## Experimental

### Fabrication of SF/SSF and PTFEF/SSF Yarns

Commercial SF (~ 150 μm in diameter), PTFEF (~ 180 μm in diameter), and SSF (~ 200 μm in diameter) were selected as raw materials to fabricate EHTs. A custom made co-wrapping spinning apparatus was used for fabricating SF/SSF yarns and PTFEF/SSF yarns. The apparatus is composed of three key parts. The first part is a feeding motor to provide the core fibers, the second is a co-wrapping system made by a rotator and a force controller, the third part is a motor-based collecting system. During the co-wrapping spinning, the wrap angle, namely wrapping density of shell fibers, can be modulated by changing the speed ratio between the drawing speed of core fiber, *v* (mm/min), and the rotating speed of shell fibers, $\omega$(rpm). The quantitative relationship among them can be predicted as Eq. 1:

1 $$\tan \alpha = \frac{{2\pi (r + D_{1} /2)\omega }}{v}$$

where $\alpha$ is the wrapping angle of shell fibers, $r$ is the radius of core fiber (SSF), *D*_1_ is the diameter of shell fibers vertical to composite yarn axis.

In addition, $\frac{\omega }{v}$ is critical for producing fully wrapped wormlike structure. According to the geometry of full-packaged core–shell structure, when wrapping angle $\alpha$ matches (Eq. 2)

2 $$\cos \alpha = \frac{{D_{2} }}{{2\pi (r + D_{1} /2) }}$$

full-packaged core–shell yarns can be obtained, where $D_{2}$ is the diameter of the shell fibers parallel to the composite yarn axis. On this condition, $\frac{\omega }{v}$ can be deduced according to Eqs. 1 and 2, that is,

3 $$\frac{\omega }{v} = \sqrt {\frac{1}{{D_{2}^{2} }} - \frac{1}{{4\pi^{2} (r + D_{1} /2)^{2} }}}$$

In other words, to produce the wormlike shell structure, $\frac{\omega }{v}$ needs to match Eq. 3. When $\frac{\omega }{v} < \sqrt {\frac{1}{{D_{2}^{2} }} - \frac{1}{{4\pi^{2} (r + D_{1} /2)^{2} }}}$, the core SSF will be partially uncovered. In contrast, when $\frac{\omega }{v} > \sqrt {\frac{1}{{D_{2}^{2} }} - \frac{1}{{4\pi^{2} (r + D_{1} /2)^{2} }}}$, the core SSF will be covered by the multilayer shell yarns. By following this criterion, in typical processing of SF/SSF yarns, the speed of wrapping unit and speed of drawing unit were fixed at 700 rpm and 120 mm min^−1^, respectively. For the PTFEF/SSF yarns, the speed of drawing unit was changed to 150 mm min^−1^ because of the larger diameter of PTFEF. The resultant SF/SSF and PTFEF/SSF yarns feature with a high helix wrapping angle (approach to 90°). The morphology of the SF/SSF and PTFEF/SSF yarns was characterized by scanning electron microscopy (SEM, JSM7800MF, JEOL).

### Fabrication of EHTs

A shuttle loom and an embroidery machine were used to weaving SF/SSF or PTFEF yarns into fabric EHTs. For the embroidery processing, the SF/SSF and PTFEF/SSF yarns were used as base yarns and a commercial polyester fiber was used as facial yarns. To fabricate the EHT-based floor tiles, two pieces of poly(methyl methacrylate) (PMMA) were shaped by a laser cutter as substrates with the dimension of 30 × 30 cm^2^. Four holes were drilled at each corner of PMMA substrates for spring installation. SF/SSF fabric and PTFEF/SSF fabric with a dimension of 20 × 20 cm^2^ were then assembled onto inner sides of two PMMA substrates. At last, four springs were anchored to connect the top and bottom substrate. To prepare the multilayer structured EHTs, four positive and negative floor tiles with sizes of 5 × 5 cm^2^ were arranged parallelly along their normal direction, and two ends of these tiles are fixed and held by Kapton films, which can serve as linkers and spacers to connect the positive and negative EHT tiles.

### Mechanical Testing of the SF/SSF Yarns

The SF/SSF yarns were first cut into 40 mm segments for tensile tests. For tensile testing, the 40 mm segments were mounted onto the testing machine (Instron 5966 machine, Instron, Norwood, MA, USA). Meanwhile, the initial length of the yarn was measured with a caliper at zero load point (the point in which the yarns are tight, but no force exerted on it). All the tensile tests were carried out at 23 °C and 75% relative humidity with a tensile speed of 0.5 mm min^−1^. For bending tests, the yarn segments with length of 40 mm were firstly mounted onto the tensile machine. A beading force was then employed on the yarn segment using compression test mode. All the bending tests were carried out at 22 °C and 70% relative humidity with a bending speed of 1 mm min^−1^.

### Electric Measurements of the EHTs

For the electric output measurement of the EHTs, a liner motor (SA-JZ010, SHIAO Company, China) was applied to mimic human motions, operating the contact and separation of the EHTs. A programmable electrometer (Keithley 6514) was adopted to test the open-circuit voltage, short-circuit current, transferred charge, and output voltage at different external load. The software platform is constructed based on LabView, which can realize real-time data acquisition control and analysis. The output performance of the EHTs was tested at relative humidity of 40%. For calculating the *J*_*sc*_, transferred charge density and power density of the EHT, the effective area (area occupied by SF/SSF or PTFEF/SSF yarns) of the 5 × 5 cm^2^ EHT was calculated by pixel statistics based on the fact that the SF/SSF or PTFEF/SSF yarns are white and the base fabrics are black. The calculated area of the EHT is about 19 cm^2^.

## Results and Discussion

### Material Screening

**Fig. 2:**
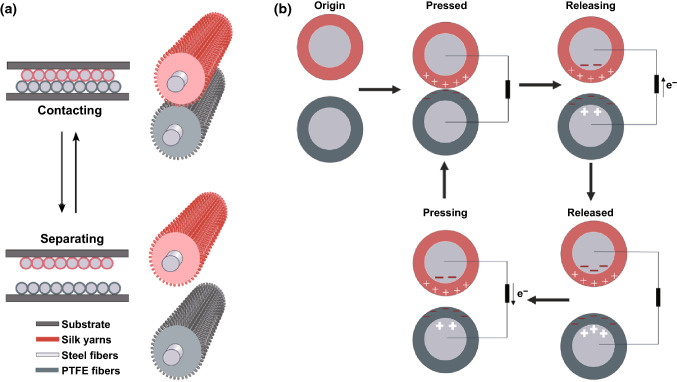
Schematic illustration and working principle of SF/PTFEF EHT: **a** schematic diagram of SF/PTFEF EHT during working. **b** schematic illustration of charge generation and transfer of contact–separation mode of SF/PTFEF EHT during contacting and separating

Compared with widely developed transfer printed and atomic deposited EHTs [2], coaxial yarn-based EHTs have advantages in scalable-manufacturing and structural stability [35]. Therefore, in this study, coaxial yarn-based EHTs were designed. Material screening is the initial and essential step in EHT design. For coaxial yarn-based EHTs, the contact–separation mode is the most widely used configuration (Fig. 2a, b), where both electrically positive and negative yarns are needed [36]. The positive yarn loses electrons during friction with other materials, whereas the negative yarn gains electrons. When these two types of yarns with different electron-attracting abilities are brought into contact, friction occurs, and then an electric potential is formed between the two interfaces. Once they are separated, an alternate potential can drive electrons in the outer circuit to flow back and forth to balance the potential. Therefore, the shell layer works as a dielectric layer, while the core layer should be highly conductive. Compared with other highly conductive materials such as copper wires, SSF has the advantages of low cost and excellent mechanical tenacity; therefore, SSF is selected as the core fiber in both positive and negative yarns.

However, the shell layer should be constructed using materials with either positive or negative triboelectric properties [37]. Almost all materials have triboelectrification activity, but their ability to gain and lose electrons is different depending on their polarity. This ability has been quantitatively evaluated by measuring the charge density produced on dielectric surfaces by contact with metals of known difference in work function *in vacuo* [38]. Hence, the positions of the most used materials can be directly found in “triboelectric series” [38–41]. In this study, SF was selected for constructing the shell layer of positive yarns because of their two intrinsic advantages. First, SF has a high dielectric property with a strong tendency to lose electrons and become positively charged when SF undergoes friction with other materials [42, 43]. Second, benefiting from unique natural hierarchical structures, SF has a high mechanical performance (with a strength of 600 MPa and toughness of 70 MJ m^−3^) [44] and excellent fracture resistance than most other fiber materials [45–48] (Fig. S1).

Once the material for positive yarns is determined, the key criterion to select the negative material is that its distance from SF in the “triboelectric series” should be large enough to ensure an adequate charge transfer during the contact-and-separate process. Meanwhile, the appropriate mechanical strength and flexibility of resulting yarns should be ensured. Herein, PTFEF was selected to construct negative yarns because it has the most different charge affinity with SF, which is particularly significant for surface charge generation on dielectric surfaces.

### Geometrical and Structural Design of Yarns for Mechanical Optimization

**Fig. 3:**
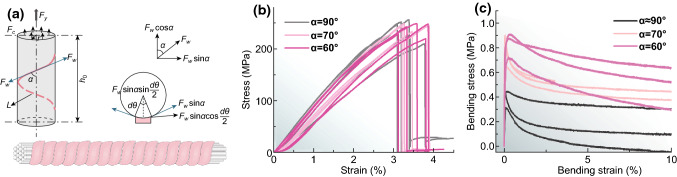
Geometrical and structural design of SF/SSF and PTFEF/SSF yarns for mechanical optimization: **a** Schematic illustration of geometry and forces acting on a warping pitch of co-wrapped yarn. **b** Stress–strain curves of SF/SSF yarns with different wrapping angles of shell SF yarns. **c** Bending stress–bending strain curves of SF/SSF yarns with different wrapping angles of shell SFs

For coaxial yarns, the helix wrapping angle ($\alpha$) controls the mechanical and triboelectric properties of yarns. Thus, in this section, $\alpha$ was designed for optimizing both mechanical and triboelectrification performance. Figure 3a shows the geometry and forces acting on a warping pitch of the co-wrapped yarn [49–52]. When the co-wrapped yarn is subjected to longitudinal force ($F_{Y}$), the force is separately shared by the warp ($F_{W}$) and core ($F_{C}$) sections. The contribution of wrapper stress to the yarn strength ($F_{S}$) can be expressed as Eq. 4 [49, 50]:

4 $$F_{S} = F_{W} \cos \alpha$$

Thus, as the wrapping density is increased, $\alpha$ is increased, and the contribution of wrapped fiber to the yarn strength is decreased. In our cases, the contribution of wrapper fibers can be ignored because SSF is much stiffer and ductile than both SF and PTFEF. SSF is the main component for providing both tensile and bending strength and hardness of yarns in both yarns (Fig. 3b). On the other hand, the component of wrapper stress, the key source of lateral force ($F_{L}$) exerted on the core SSF, can be dramatically increased with the increase in $\alpha$ with a quantitative relationship that can be expressed as Eq. 5:

5 $$F_{L} = F_{w} \sin \alpha \sin \frac{{{\text{d}}\theta }}{2}$$

According to this equation, the wrapper stress can significantly reduce the freedom of SSF movement in yarn core, and thereby the core SSF can be contacted more closely than the yarns with a smaller $\alpha$.

Notably, the flexural rigidity of yarns is considerably higher than those of ply or parallel yarns without a wrapper layer [53]. The increase in flexural rigidity obtained by wrapping can be estimated from the factor $\frac{N}{y}$, where $N$ is the number of SSFs in the cross section and $y$ is the yarn density or fiber density [54]. This indicates that for co-wrapped yarns with no freedom of fiber movement, the flexural rigidity should be about 200 times as large as that of parallel yarns. The increase in flexural rigidity has two distinct effects on yarn processability. On the one hand, the high flexural rigidity can improve the ability of yarns against the deformation; on the other hand, it has a negative impact on machine weaving. Therefore, an appropriate flexural rigidity is desired for the yarns. For coaxial yarns, this trade-off can be achieved by $\alpha$ increasing. According to Backer’s theory [55], the high twisted co-wrapped yarns have a lower flexural rigidity than low twisted yarns (Fig. 3c). Based on these mechanical assessments, both positive and negative yarns were designed as wormlike core–shell structure with a high helix wrapping angle of ~ 90° (Fig. 3a). In practice, such wormlike co-warping structures indeed have been widely used in friction tolerance applications such as music instrument strings; they are often designed as a “core” of one material and an over winding of another material. This structure can make the string vibrate at the desired pitch while maintaining a low profile and sufficient flexibility for playability.

### Structural Design of Yarns for the Modulation of Triboelectrification Performance

**Fig. 4:**
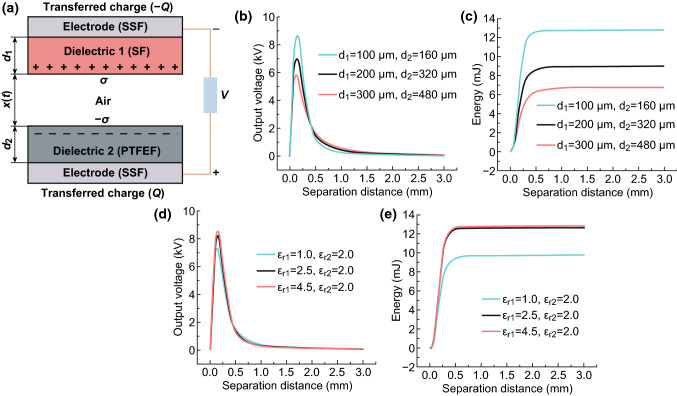
Structure of EHT theoretical model and effect of thickness and relative permittivity on real-time output voltage and energy: **a** Simplified structure of SF/PTFEF EHT. **b**, **c** Influence of thickness of SF (*d*_1_) and PTFEF (*d*_2_) on the output voltage and energy of SF/PTFEF EHT, respectively. **d**, **e** Influence of relative permittivity of SF (*ε*_*r*1_) on the output voltage and energy of SF/PTFEF EHT, respectively

As mentioned, SF/PTFEF EHTs generate voltage through the contact–separation mode; therefore, the thickness of shell layers ($d$) is important in controlling the triboelectrification performance (i.e., output energy $E$) of the final system. To quantitatively evaluate these effects, SF/PTFEF EHTs were simplified into the contact–separation mode in plane (Fig. 4a) [56]. In this mode, the inner SSF constitutes the two electrodes, whereas cortical SF and PTFEF are positive (dielectric 1) and negative dielectric (dielectric 2), respectively. When the dielectrics layers were contacted with each other by mechanical force, opposite and equal amount of $\sigma$ is generated on the surface of two triboelectric layers due to electrostatic induction. For dielectrics, it is reasonable to assume that the electrostatic charge is evenly distributed on both surfaces, and the electrostatic charge could stay for a long time [57, 58]. When the separation distance starts to increase under the mechanical force, an alternative potential difference between the two electrodes, i.e., output voltage $V$, is induced. The output voltage ($V$) can be evaluated using $V{-}Q{-}x$ equation and expressed as Eq. 6 [59]:

6 $$V(t) = R\frac{{{\text{d}}Q}}{{{\text{d}}t}} = - \frac{Q}{{S\varepsilon_{0} }}\left( {\frac{{d_{1} }}{{\varepsilon_{r1} }} + \frac{{d_{2} }}{{\varepsilon_{r2} }} + x(t)} \right) + \frac{\sigma x(t)}{{\varepsilon_{0} }}$$

where $S$ is the contact area of two shell layers during working, $\varepsilon_{r}$ is the relative permittivity of dielectric layers, $\varepsilon_{r0}$ is the vacuum permittivity, $\sigma$ is the electrostatic charge density, $Q$ is the amount of transferred charge between electrode layers, $x$ is the separation distance between the two dielectric layers. The potential difference between two sides of the EHT can drive electrons in the outer circuit to move directionally. As a result, when dielectric 1 and 2 layers were periodically driven to contact and separate with each other at a constant velocity $(v )$, the real-time output energy ($E$) can be evaluated by solving the differential of Eq. 6. The detailed derivation process is given in Supporting Information. The resulting equation can be expressed as Eq. 7:

7 $$E = \mathop \smallint \limits_{0}^{{\frac{{x_{\text{max} } }}{v}}} \frac{{V(t)^{2} }}{R}{\text{d}}t = \frac{1}{v}\mathop \smallint \limits_{0}^{{x_{\text{max} } }} \frac{{V(x)^{2} }}{R}{\text{d}}x$$

By substituting the empirical parameters (as listed in Table S1) into these equations, the effect of thickness and relative permittivity of shell SF/PTFEF yarns can be directly predicted on real-time output voltage and energy of SF/PTFEF EHT (Fig. 4b–e). As shown in Fig. 4b, c, output voltage peak (*V*) and output energy (*E*) of EHT decrease with the increase in thickness of SF and PTFEF layers. Same relationship can be also seen between transferred charge ($Q$) and thickness of shell layers (Fig. S2a), indicating that the thinner shell layer is better for achieving a high triboelectrification performance. Hence, in EHT fabrication, a single shell layer of SF or PTFEF was wrapped around the SSF to promote the elegant balance of lightweight, mechanical, and triboelectric properties.

### Modified Rotor Co-wrapping Spinning for the Fabrication of Wormlike Yarns

To produce wormlike coaxial yarns made of two types of continuous filaments, rotor co-wrapping spinning techniques (RCWSTs) are used. A series of advanced yarns (known as the fifth generation of yarns) can be fabricated using RCWSTs, such as technical yarns, hybrid yarns, and conductive yarns [60]. However, the commercial RCWSTs are not suitable for this study because they are difficult to spin hybrid yarns with a high α angle, especially yarns with no gaps between two pitches. Therefore, we designed a rational RSWST for spinning wormlike positive SF/SSF and negative PTFEF/SSF yarns based on the rotor co-wrapping principle.

**Fig. 5:**
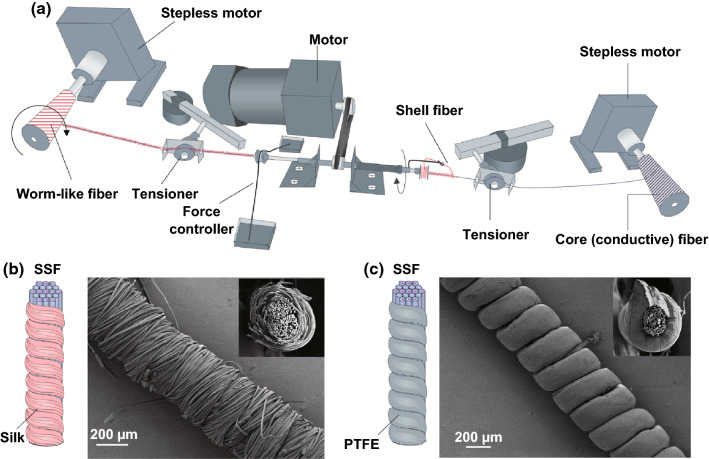
Fabrication of SF/SSF and PTFEF/SSF yarns: **a** schematic diagram of the apparatus for fabricating SF/SSF and PTFEF/SSF yarns. Schematic illustration and SEM image of **b** SF/SSF yarns and **c** PTFEF/SSF yarns. The insets are SEM image of cross section of these yarns

As shown in Fig. 5a and Video S1, during the rotor co-wrapping spinning, the SSF was continuously passed through the hollow tube with a constant rate, which is controlled by the drawing rollers. At the same time, a metal rotor loaded with SF or PTFEF rotated counterclockwise, so that the SF/PTFEF were wrapped on the SSFs to produce the final yarns. In this process, the wrap angle can be tailored by changing the drawing speed ratio between the drawing speed of core fiber and the rotating speed of wrapper fibers to obtain different wrapping density of core–shell yarns (Fig. S3). To achieve the fully package of shell yarns (namely no gap exists between two adjacent wrapping fibers), the ratio between the wrapping speed ($\omega$, rpm) and the drawing speed of core fiber (*v*, mm min^−1^) needs to match Eq. 8:

8 $$\frac{\omega }{v} = \sqrt {\frac{1}{{D_{2}^{2} }} - \frac{1}{{4\pi^{2} (r + D_{1} /2)^{2} }}}$$

where $r$ is the radius of core fiber (SSF), *D*_1_ is the diameter of shell fibers vertical to composite yarn axis, $D_{2}$ is the diameter of the shell fibers parallel to composite yarn axis. The detailed derivation for such equation can be found in Experimental Section. The speed ratio of $\frac{\omega }{v}$ thus can directly obtained by substituting the diameters of the shell SF/PTFEF and core SSF (Table S2) into Eq. 8. Following this criterion, in co-wrapping spinning, the speed of wrapping unit and speed of drawing unit were fixed at 700 rpm and 120 mm min^−1^, respectively. Under these conditions, predesigned SF/SSF yarns can be generated (Fig. S4a) with a length of 7.2 m per hour. For the negative PTFEF/SSF yarns (Fig. S4b), the speed of drawing unit, instead, was changed to 150 mm min^−1^ due to a larger diameter of PTFEF (Table S2).

### Structure and Mechanical Properties of Wormlike Yarns

Figures 5b, c show the structures of a SF/SSF yarn and PTFEF/SSF yarn spun with ~ 5800 and ~ 4700 turns/m, respectively. In both the yarns, the wrapping fibers fully cover the SSF core with no gaps between two pitches. However, careful examination of the surface of SF layer showed the presence of micro-interstices between SFs. This often results in the decrease in relative permittivity due to the existence of air in these micro-interstices. As evaluated from Eq. 7, by decreasing the relative permittivity of SF layer from 4.5 to 2.5, the output voltage of the entire system decreases by 3.9% (Fig. 4d), output energy decreases by 1.4% (Fig. 4e), and transferred charge decreases by 0.6% (Fig. S2b), respectively. An SF welding strategy can be applied to solve this problem. Specifically, hexafluoroisopropanol (HFIP) can be used to selectively etch the surface of SF, and the silk proteins thereby can act as a solid glue to fill the microinterstices and to obtain a fully covered SF dielectric layer (Fig. S5).

SF/SSF yarns have a tensile strength of 237 ± 13 MPa and toughness of 4.5 ± 0.4 MJ m^−3^ (Fig. 3b), comparable with other high-performance functional yarns such as MWCNT-glass fiber [61] and graphene fiber [62]. More importantly, no significant mechanical differences were observed between wormlike yarns and parallel SSFs (without a wrapping layer, Fig. S6), confirming that the core SSF indeed controls the mechanical strength of yarns. The cross-sectional scanning electron microscopy (SEM) images verify that the core SSF is intensively contacted due to the high lateral force contributed by the wrapped fibers (insert in Fig. 5b, c). This makes the core fiber bundles remain compact even when the yarns undergo tensile failure (Fig. S7).

**Fig. 6:**
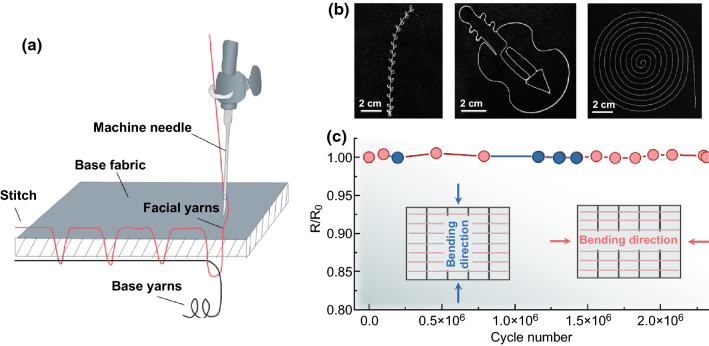
Mechanized fabrication of SF/SSF and PTFEF/SSF textiles: **a** sketch map of the embroidery machine weaving yarns into fabric. **b** Different patterns of fabricated SF/SSF and PTFEF/SSF fabrics obtained using embroidery machine. **c** Resistance changes of the SSF in SF/SSF fabric during longtime cyclic deformations. The insets are diagrams of bending direction of SF/SSF textiles during deformations (bending direction along with the arrows)

The mechanical advantages of these yarns support their diverse processability. They can be directly weaved into any desired pattern using an automatic embroidery machine (Fig. 6a, b). SEM images of the pattern (Fig. S8) show that the yarns were intact and bound with the substrate fabric well. These yarns can also be weaved into large-scale textile using a loom, enabling the applications of these textiles in non-apparel fabric (Fig. S9). Because of the ultrastable yarn structure, the resulting textile shows outstanding tolerance to multi-type fierce deformations. As shown in Figs. 6c and S10, SF/SSF textile weaved by embroidery machine exhibits almost no change in conductivity and appearance after millions of cyclic deformations along the longitudinal and transverse directions (Video S2), superior than most conductive wires. In addition, SEM image of the SF/SSF yarn has undergone 2.3 million bends shows that the SF still firmly winds on the SSF (Fig. S10f), which is essential to achieve long-term wearability.

### Preparation and Performance of SF/PTFEF EHTs

**Fig. 7:**
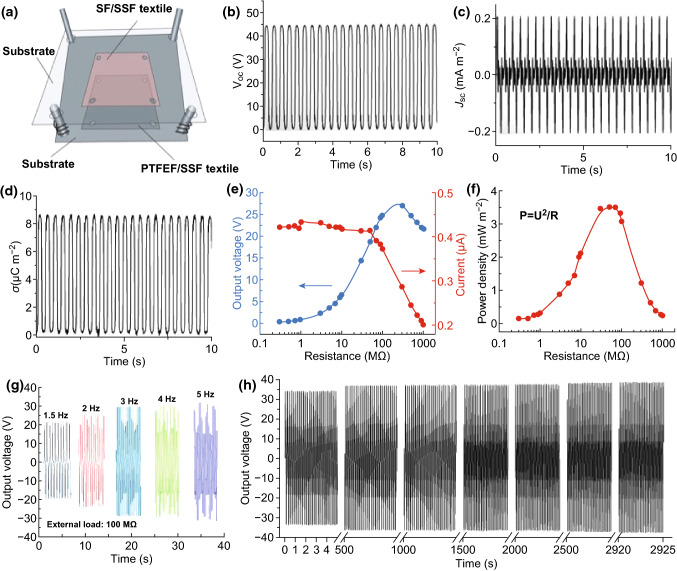
EHT and its electrical output characterization: **a** Schematic illustration of self-powered EHTs. **b**–**d** The open-circuit voltage (*V*_oc_), short-circuit current density (*J*_sc_), and transferred charge density (*σ*) of EHT under an operation frequency of 2 Hz, respectively. **e** Dependence of current and voltage output of EHTs on the external loading resistance. **f** Dependence of power densities of EHT on the external load resistance. **g** Measured output voltage of EHTs on the external loading of 100 MΩ at different work frequencies. **h** Stability and reliability of SF/PTFEF EHT measurements, where the voltage was recorded for 14,000 cycles on the external loading of 100 MΩ and work frequency of 5 Hz

The output performance of SF/PTFEF EHT was tested using a device shown in Fig. 7a. Two pieces of fabric with the same dimension (5 × 5 cm^2^) made of SF/SSF and PTFEF/SSF yarns were placed on the upper and lower sides of polymethyl methacrylate plates, respectively. Then, four springs were anchored to connect the bottom and top substrates for assisting in the separation of two substrates. Figure 7b–f shows the typical electrical signals of EHTs. When the SF/SSF and PTFEF/SSF textiles were periodically pressed to contact with each other at a frequency of 2 Hz, an open-circuit voltage (*V*_oc_) of ~ 45 V (Fig. 7b), a short-circuit current density (*J*_sc_) of ~ 0.2 mA m^−2^ (Fig. 7c), and a transferred charge density of ~ 8.6 mC m^−2^ (Fig. 7d) were obtained.

The dependence of current and voltage output of EHTs on the external loading resistance was also evaluated. As shown in Fig. 7e, with the increase in load resistance, the voltage showed an increasing trend, whereas the current exhibited a reducing tendency as a result of ohmic loss [63]. The output voltage and output current peak can reach up to 27 V and 0.43 μA, respectively. The maximum power density at a resistance of 50 MΩ was ~ 3.5 mW m^−2^ (Fig. 7f). The generated output power is enough to reduce the power consumption of some electronics, thus effectively addressing the big concern of sustainable power supply for wearable systems reliably. Moreover, the output voltage peak of EHTs on the external loading resistance can be increased by increasing the working frequency. As shown in Fig. 7g, the output voltage peak of EHTs on the external loading of 100 MΩ increased from 22 to 32 V when the work frequency increased from 1.5 to 5 Hz. The relation between the voltage and the transfer time (or frequency) in this condition can be estimated through the equation of *V *= *QR/t*. Here, the transfer charge (*Q*) generated by each contact with different frequency can be regarded as a constant since the area in each contact is almost the same. Accordingly, this equation reveals that the higher working frequency [namely less charge transfer time (*t*)] can result in faster charge transfer between two electrodes of the SF/PTFEF EHT and thereby lead to the increase in the output voltage on a certain external load. Figure 7h shows good stability of EHTs validated by a 14,000-cycling test (working frequency: 5 Hz). This is particularly important for practicality in wearable fabrics. Although the output performance of SF/PTFEF EHT is inferior to those TENGs feature with nanostructures on the dielectric surface and ultrahigh charge density [64, 65], SF/PTFEF EHT is comparable with other fabric TENGs [16, 18, 30]. Further, the power density can be enhanced by increasing the layers of SF/SSF and PTFEF/SSF textiles to fabricate 3D structure EHTs [12].

### Potential Applications of SF/PTFEF EHTs

**Fig. 8:**
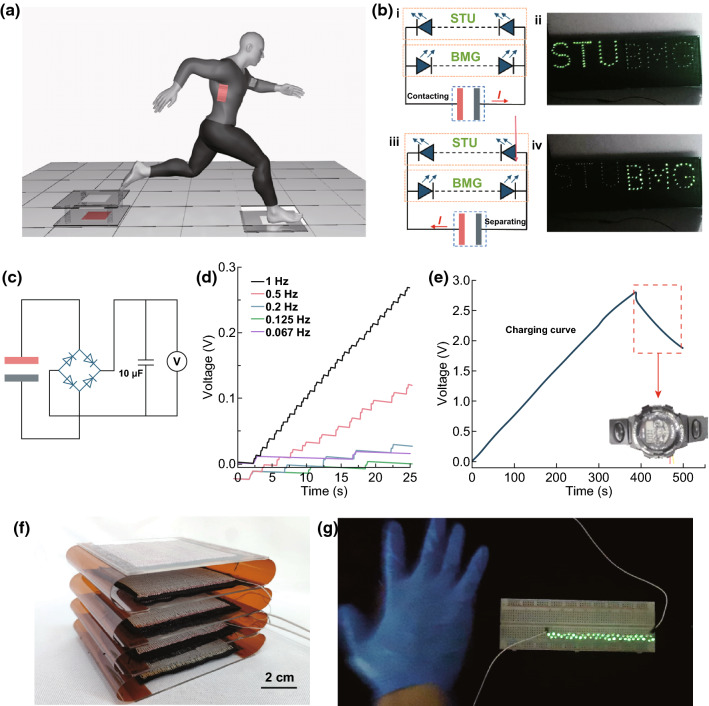
Applications of EHTs: **a** Schematic illustration of the use of EHTs as wearable power generation fabrics and floors to harvest energy from human motion. **b** (i) Schematic diagram of connection between the energy harvesting floor and LEDs shaped into the letters “STU” and “BMG.” (ii) Photograph of the energy harvesting floor driving the LEDs shaped into the letters “STU.” (iii) Schematic diagram of the reversed connection between the energy harvesting floor and LEDs that make up the letters “STU” and “BMG.” (iv) Photograph of the LEDs shaped into the letters “BMG” lighted up by the reversely connected energy harvesting floor. **c** Circuit diagram of the energy harvesting floor to continuously charge a capacitor of 10 µF with a rectifier. **d** Measured voltage of a 10-µF capacitor charged by the energy harvesting floor at different frequencies. **e** Charging curve of a 100-µF capacitor charged by the energy harvesting floor at a frequency of 5 Hz. The inset shows the photograph of charged capacitor to power an electronic watch. **f** Photograph of multilayered EHTs with four-unit numbers connected in parallel. **g** Photograph of 46 green LEDs connected in parallel powered by the resulting multilayered SF/PTFEF EHTs (ambient humidity: **b** at 50%, **d**, **e** at 37%, and **g** at 65%). (Color figure online)

The mechanical advantages and mass production of EHTs allow them to be used as wearable power generation fabrics (Fig. S11a and Video S3) and large-scale energy harvesting devices such as energy harvesting floors (Fig. 8a and S11b). To demonstrate the capability of EHTs as a power source for power electronics, 106 commercial light-emitting diodes (LEDs) were connected to the two electrodes of EHTs. The LEDs were divided into two groups: One group was connected in series to the shape of letters “STU.” Another group was also connected in series but to the shape of letters “BMG.” Then, these two groups of LEDs were connected to the device with opposite polarity. The aforementioned EHT-based floor tiles with an active area of 20 × 20 cm^2^ were used. As shown in Fig. 8bi, bii), when stepping on the floor, the “STU” of 53 LEDs was lighted up. Once the foot was removed from the floor, another group of 53 LEDs “BMG” would light up, connected to the EHT-based floor tiles in reverse (Fig. 8biii, biv; Video S4).

Except for using a direct power source to power electronics, the electric energy produced by the EHT can also be stored in capacitors using a rectifier. Figure 8c shows the circuit diagram of charging a 10-μF capacitor at different frequencies. The measured voltage of the capacitor is shown in Fig. 8d, indicating that the charging rate can be increased at higher working frequencies. The EHT-based floor tiles were also tested to charge capacitors with capacitance from 10 to 330 μF at a frequency of 1 Hz (Fig. S12). In a practical application, the power generation floor was integrated with a 100-μF capacitor and an electronic watch to form a self-powered system. As shown in Fig. 8e, the 100-μF capacitor can be charged from 0 to 2.7 V in about 400 s by an EHT-based floor (charging frequency 5 Hz). The stored energy could continuously drive an electronic watch working for ~ 100 s (inset in Fig. 8e). Benefitting from its easy processing, several EHT units (typically four) can be integrated together and connected in parallel to enable high-power energy harvesting. Figure 8f shows the photograph of a multilayered EHT with four 5 × 5 cm^2^ units. This fabricated multilayered EHT easily lighted up 46 LEDs with one tap by hand (Fig. 8g).

## Conclusions

In this work, a de novo design strategy is proposed for advanced energy textiles, start with material screening, geometrical and structural design, modulation of triboelectrifications and automatic fabrication process, down to evaluation and optimization of the mechanical and triboelectric performance, and finally reach to the practical applications. Different from other EHT systems which commonly introduce functions to the existed textiles, this work aims to develop functional yarns which can tolerate mechanized processing and long-term use. Following with theoretical analysis from both mechanical and electric aspects, a core–shell structure was finally selected to construct triboelectric yarns. Such a predesigned structure provides the possibility to balance the mechanical and triboelectric performance of the resultant EHT system and therefore allow to maintain EHT’s high energy output and structural stability in practice long-term use, considering the merits of SF/PTFEF EHTs in processability and long durability to explore broader application prospects, including wearable electronics, motion tracking, artificial intelligence, and human-interactive interfaces.

## Electronic supplementary material

Below is the link to the electronic supplementary material.

Supplementary material 1 (PDF 980 kb)

Supplementary material 2 (MP4 6933 kb)

Supplementary material 3 (MP4 10096 kb)

Supplementary material 4 (MP4 3381 kb)

Supplementary material 5 (MP4 10,066 kb)
