# Correction to: Ultrastable and High-Performance Silk Energy Harvesting Textiles

**DOI:** 10.1007/s40820-020-0380-z

**Published:** 2020-01-30

**Authors:** Chao Ye, Shaojun Dong, Jing Ren, Shengjie Ling

**Affiliations:** School of Physical Science and Technology, ShanghaiTech University, 393 Middle Huaxia Road, Shanghai, 201210 People’s Republic of China

## Correction to: Nano-Micro Lett. (2020) 12:12 10.1007/s40820-019-0348-z

In the original publication, there is line dislocation in Fig. 8d. The correct Fig. 8 is provided in this correction.

**Fig. 8:**
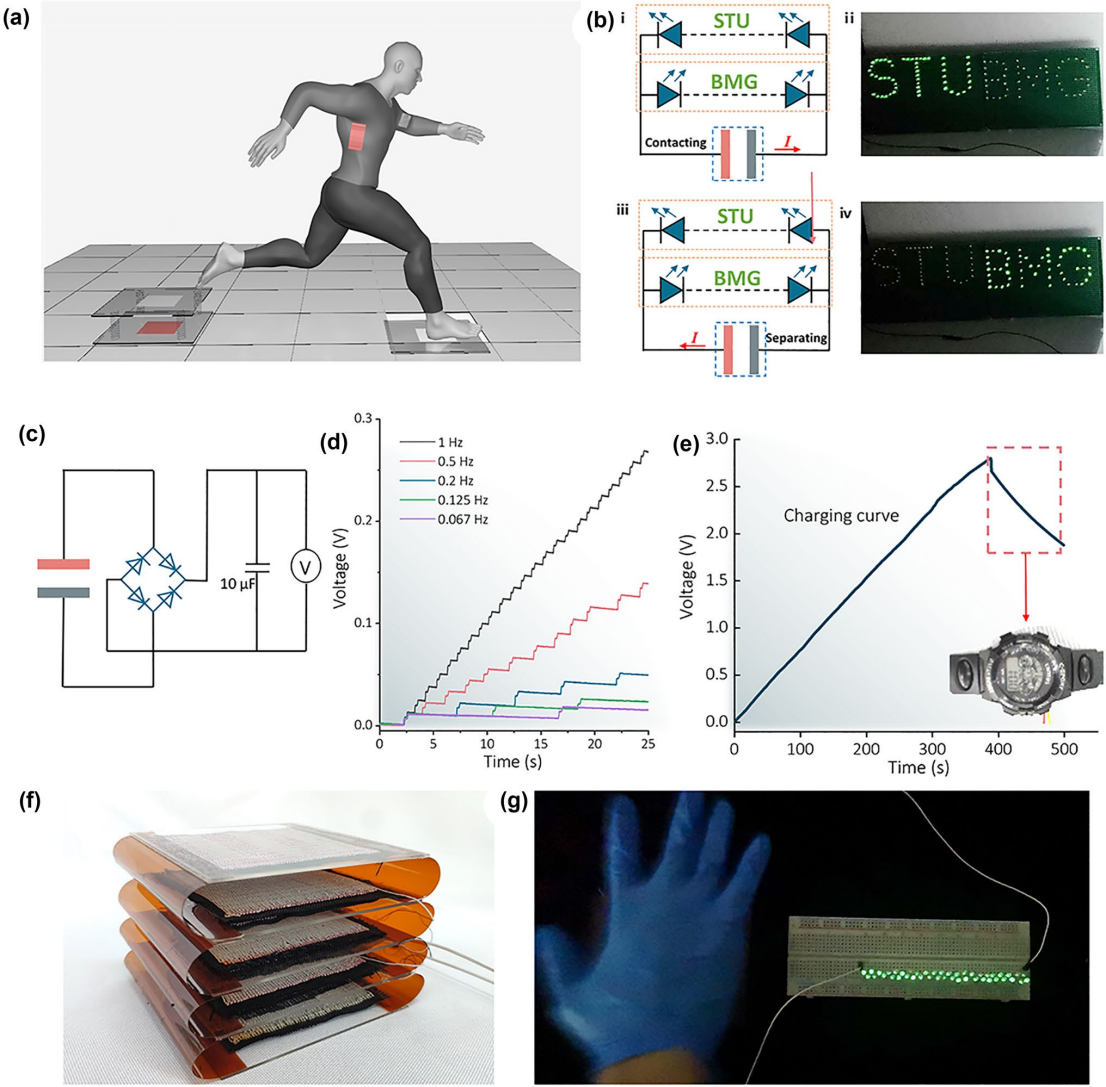
Applications of EHTs: **a** Schematic illustration of the use of EHTs as wearable power generation fabrics and floors to harvest energy from human motion. **b** (i) Schematic diagram of connection between the energy harvesting floor and LEDs shaped into the letters “STU” and “BMG.” (ii) Photograph of the energy harvesting floor driving the LEDs shaped into the letters “STU.” (iii) Schematic diagram of the reversed connection between the energy harvesting floor and LEDs that make up the letters “STU” and “BMG.” (iv) Photograph of the LEDs shaped into the letters “BMG” lighted up by the reversely connected energy harvesting floor. **c** Circuit diagram of the energy harvesting floor to continuously charge a capacitor of 10 μF with a rectifier. **d** Measured voltage of a 10-μF capacitor charged by the energy harvesting floor at different frequencies. **e** Charging curve of a 100-μF capacitor charged by the energy harvesting floor at a frequency of 5 Hz. The inset shows the photograph of charged capacitor to power an electronic watch. **f** Photograph of multilayered EHTs with four-unit numbers connected in parallel. **g** Photograph of 46 green LEDs connected in parallel powered by the resulting multilayered SF/PTFEF EHTs (ambient humidity: **b** at 50%, **d**, **e** at 37%, and **g** at 65%) (Color figure online)

