# Supplementary material for: Ultrastable and High-Performance Silk Energy Harvesting Textiles
Source: Nanomicro Lett. 2019 Dec 30;12:12. doi: 10.1007/s40820-019-0348-z (PMC7770758; doi:10.1007/s40820-019-0348-z)
Supplement: Supplementary file 1 — Supplementary material 1 (PDF 980 kb) [file 40820_2019_348_MOESM1_ESM.pdf]

Supporting Information for

Ultrastable and High Performance Silk Energy Harvesting Textiles

Chao Ye[1], Shaojun Dong[1], Jing Ren[1,*], Shengjie Ling[1,*]

[1]School of Physical Science and Technology, ShanghaiTech University, 393 Middle Huaxia Road, Shanghai 201210, People's Republic of China

*Corresponding authors. E-mail: renjing@shanghaitech.edu.cn (Jing Ren); lingshj@shanghaitech.edu.cn (Shengjie Ling)

Supplementary Figures and Tables

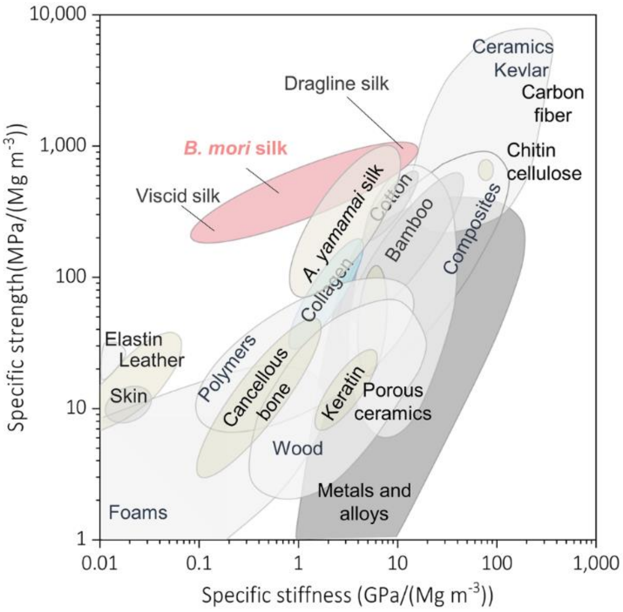

Fig. S1 Comparison of the specific strength and specific stiffness of *B. mori* silk with other natural and synthetic materials. Ashby plot of natural and synthetic materials are adapted from reference [S1]

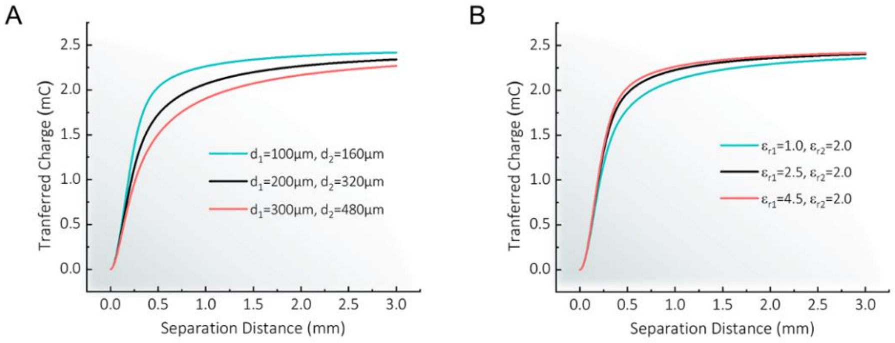

Fig. S2 Effect of thickness and relative permittivity on real-time transferred charge: **a** Effect of thickness of SF (d_1) and PTFEF (d_2) on the transferred charge (Q) of the EHT, respectively. **b** Effect of relative permittivity of SF (ε_{r1}) on the transferred charge (Q) of the EHT

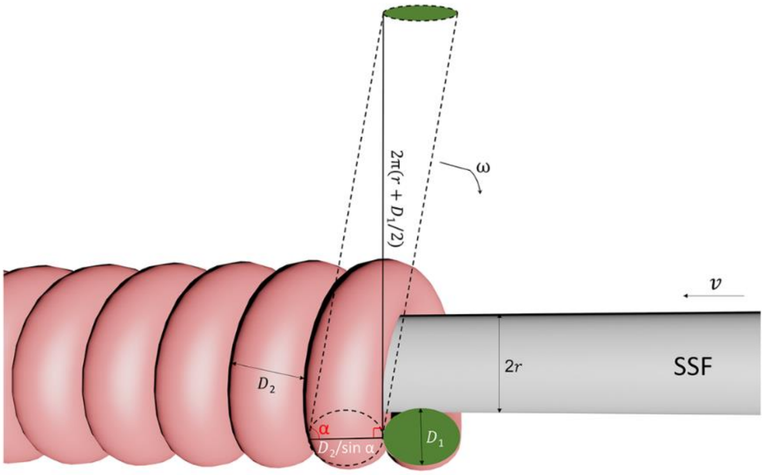

Fig. S3 Schematic illustration of geometry of full-packaged core-shell yarns without gaps on core yarns

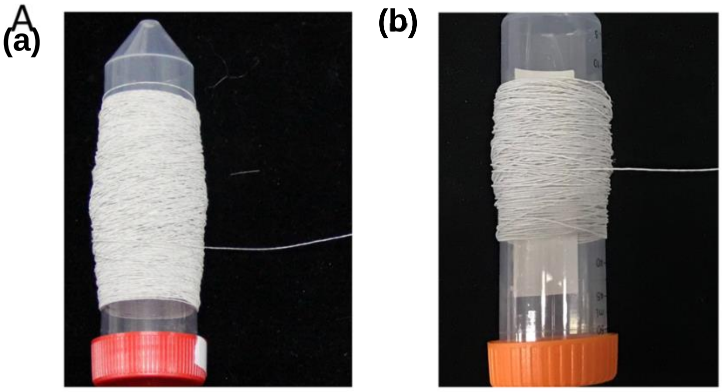

Fig. S4 Photographs of SF/SSF yarn **a** and PTFE/SSF yarn **b**

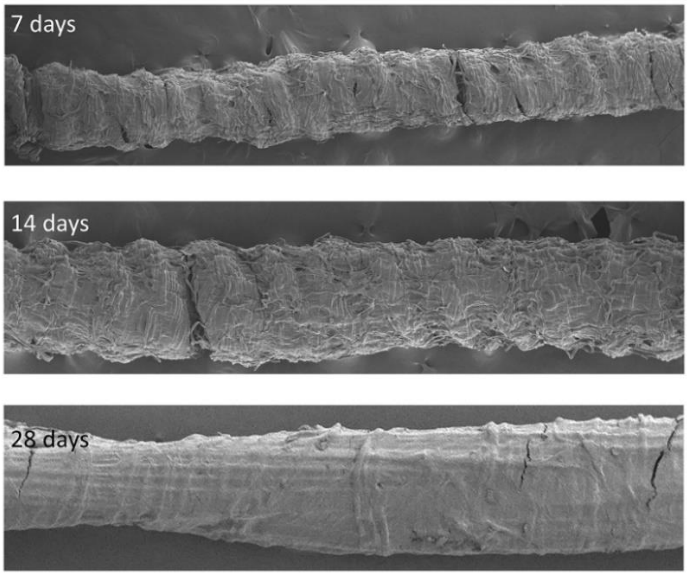

Fig. S5 SEM image of surface morphology on SF/SSF yarn after incubated in HFIP for 7days, 14days, and 28 days

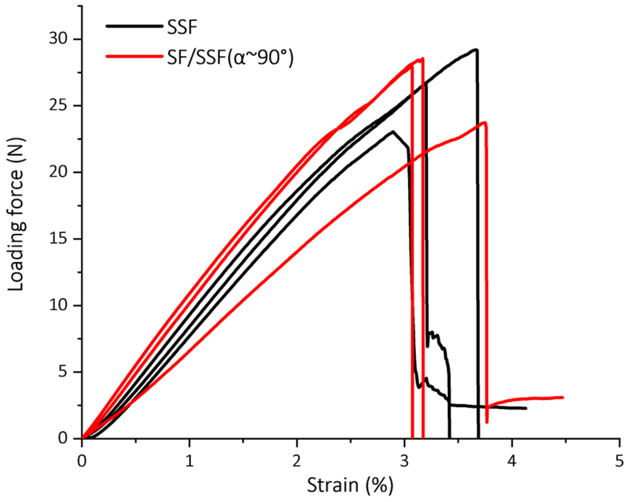

Fig. S6 Loading force-strain curves of SSFs and SF/SSF yarns

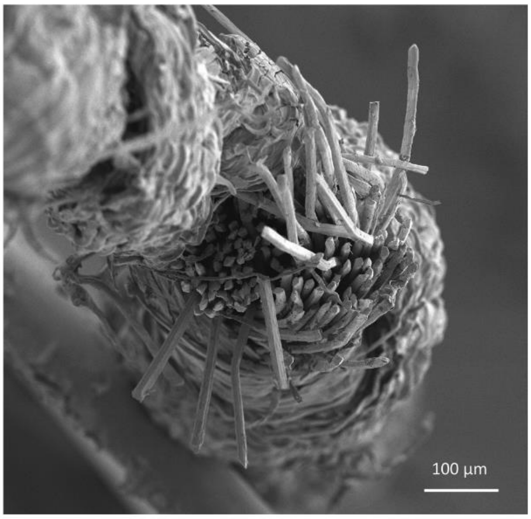

Fig. S7 SEM image of cross section of the SF/SSF yarn after being tensiled to failure

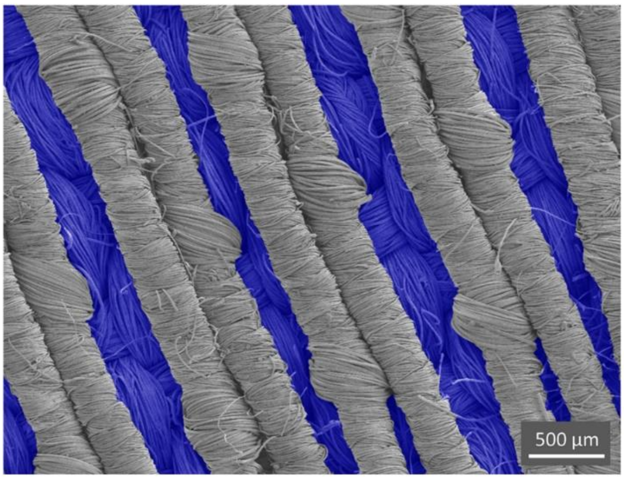

Fig. S8 SEM image of the SF/SSF yarn in EHT. The area recoloured in blue were the base fabric

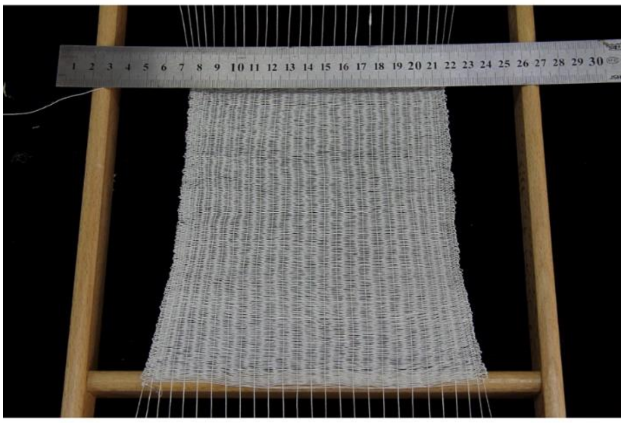

Fig. S9 Photograph of the SF/SSF yarn woven into large scale textile

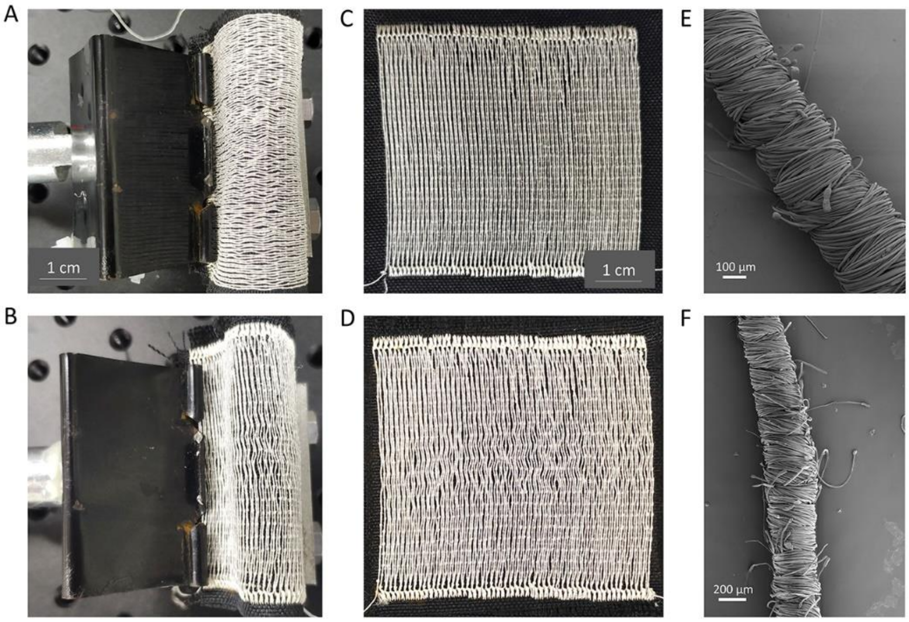

Fig. S10 Long-time cyclic deformation test of the SF/SSF fabric: a Photographs of the SF/SSF fabric bending along longitudinal directions. b Photograph of the SF/SSF fabric bending along transverse directions. c Photograph of pristine SF/SSF fabric. d Photograph of SF/SSF fabric after 2.3 million of cyclic deformations. e SEM image of pristine SF/SSF yarns. f SEM image of SF/SSF yarn after 2.3 million of cyclic deformations. image of SF/SSF

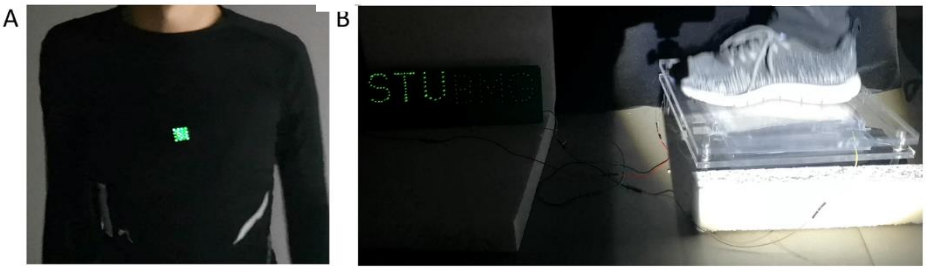

Fig. S11 a Photograph of the 8×8 cm EHTs used as wearable power generation fabric to drive 25 LEDs. (Ambient humidity: 70%) **b** Photograph of the power generation floor used to drive a group of LEDs. (Ambient humidity: 50%)

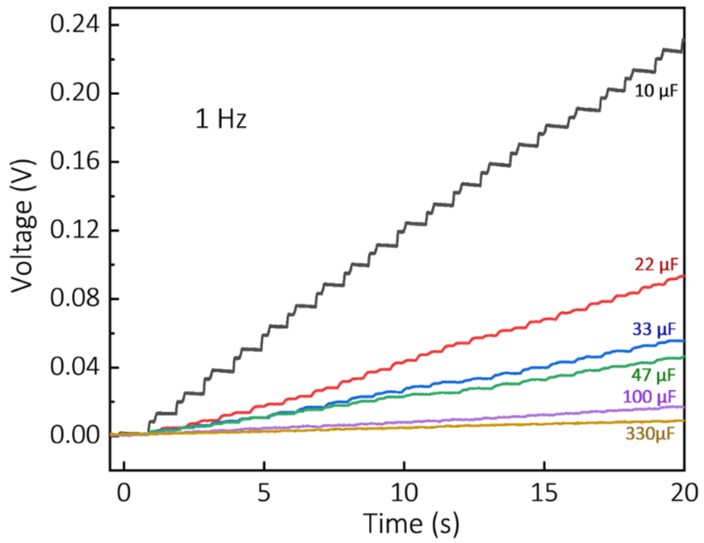

Fig. S12 Measured voltage of capacitors with different capacities (10 μF—330 μF) charged by the energy harvesting floor. (Ambient humidity: 37%)

The detailed derivation process about output voltage of SF/PTFEF EHT

The derivation is based on reference [S2]. The Equation 1 can be solved by specifying the boundary condition. We assume that the two plates are close to each other at t =0. Hence, the boundary condition is

$$Q(t=0)=0 \qquad (S1)$$

Then Eq. S1 can be solved analytically as

$$Q(t)=\sigma S-\sigma S exp\left[-\frac{1}{RS\varepsilon_0}\left(d_0t+\int_0^t x(t)dt\right)\right] -\frac{\sigma d_0}{R\varepsilon_0}exp\left[-\frac{1}{RS\varepsilon_0}\left(d_0t+\int_0^t x(t)dt\right)\right] \times\int_0^t exp\left[\frac{1}{RS\varepsilon_0}\left(d_0z+\int_0^z x(z)dz\right)\right]dz \qquad (S2)$$

Then, we consider a special case that the top plate starts to separate from the bottom one at a uniform velocity, under the external mechanical force.

$$x(t)=vt \qquad (S3)$$

Substitute Eq. S3 into Eq. S2, we can obtain,

$$Q(t)=\sigma S\left[1-exp(-At-Bt^2)+\sqrt{2}Fexp(-At-Bt^2)\times Dawson\left(\frac{F}{\sqrt{2}}\right) -\sqrt{2}F\times Dawson\left(\frac{F}{\sqrt{2}}+\sqrt{B}t\right)\right] \qquad (S4)$$

Therefore, the current and voltage output can be derived as

$$I(t)=\frac{dQ}{dt}=\sigma S\Bigg[exp(-A\mathrm{t}-\mathrm{B}t^2)(A+2\mathrm{Bt}) - \sqrt{2}Fexp(-A\mathrm{t}-\mathrm{B}t^2)(A+2\mathrm{Bt})\times Dawson\left(\frac{F}{\sqrt{2}}\right)-A + 2A\left(\frac{F}{\sqrt{2}}+\sqrt{B}t\right) \times Dawson\left(\frac{F}{\sqrt{2}}+\sqrt{B}t\right)\Bigg] \quad (S5)$$

$$V(t)=RI(t)=R\sigma S\Bigg[exp(-A\mathrm{t}-\mathrm{B}t^2)(A+2\mathrm{Bt}) - \sqrt{2}Fexp(-A\mathrm{t}-\mathrm{B}t^2)(A+2\mathrm{Bt})\times Dawson\left(\frac{F}{\sqrt{2}}\right)-A + 2A\left(\frac{F}{\sqrt{2}}+\sqrt{B}t\right) \times Dawson\left(\frac{F}{\sqrt{2}}+\sqrt{B}t\right)\Bigg] \quad (S6)$$

$$V(x)=R\sigma S\left\{exp\left(-\frac{A}{v}x-\frac{B}{v^2}x^2\right)\left(A+\frac{2B}{v}x\right)\left(1-\sqrt{2}F\times Dawson\left(\frac{F}{\sqrt{2}}\right)\right) - A\left[1-2\left(\frac{F}{\sqrt{2}}+\frac{\sqrt{B}}{v}x\right)\times Dawson\left(\frac{F}{\sqrt{2}}+\frac{\sqrt{B}}{v}x\right)\right]\right\} \quad (S7)$$

In the above expressions,

$$\mathrm{A}=\frac{d_0}{RS\varepsilon_0} \quad (S8)$$

$$\mathrm{B}=\frac{v}{2RS\varepsilon_0} \quad (S9)$$

$$F=\frac{A}{\sqrt{2B}}=\frac{d_0}{\sqrt{RS\varepsilon_0 v}} \quad (S10)$$

$$Dawson(x)=exp(-x^2)\int_0^x exp(y^2)dy \quad (S11)$$

Table S1 Parameters of the EHT

Dielectric 1 (SF)	$d_1=0.1$ mm, $\varepsilon_{r1}=4.5$
Dielectric 2 (PTFEF)	$d_2=0.16$ mm, $\varepsilon_{r2}=2$
Area size of the Dielectrics S	$25\ cm^2$
Tribo-charge surface density σ	$0.001\ C/m^2$
Maximum separation distance x_{max}	$3\ mm$
Average velocity v	$0.012\ m/s$

Table S2 Diameters of shell yarns on SSF

SF	$D_1 = 0.10mm$	$D_2 = 0.17mm$
PTFEF	$D_1 = 0.16mm$	$D_2 = 0.20mm$

Supplementary References

[S1]U.G.K. Wegst, M.F. Ashby, The mechanical efficiency of natural materials. Philos. **84**, 2167-2186 (2004). https://doi.org/10.1080/14786430410001680935

[S2]S. Niu, S. Wang, L. Lin, Y. Liu, Y. Zhou, Y. Hu, Z.L. Wang, Theoretical study of contact-mode triboelectric nanogenerators as an effective power source. Energy Environ. Sci. **6**, 3576-3583 (2013). https://doi.org/10.1039/c3ee42571a
